# Feasibility of absolute quantification for ^31^P MRS at 7 T

**DOI:** 10.1002/mrm.27729

**Published:** 2019-03-20

**Authors:** Lucian A. B. Purvis, Ladislav Valkovič, Matthew D. Robson, Christopher T. Rodgers

**Affiliations:** ^1^ Oxford Centre for Clinical Magnetic Resonance Research University of Oxford, John Radcliffe Hospital Oxford United Kingdom; ^2^ Department of Imaging Methods, Institute of Measurement Science Slovak Academy of Sciences Bratislava Slovakia; ^3^ Wolfson Brain Imaging Centre University of Cambridge Cambridge United Kingdom

**Keywords:** 7 T, absolute quantification, human, liver, ^31^P MRS, phosphorus magnetic resonance spectroscopy

## Abstract

**Purpose:**

Phosphorus spectroscopy can differentiate among liver disease stages and types. To quantify absolute concentrations of phosphorus metabolites, sensitivity calibration and transmit field (${\text{B}}_{1}^{+}$) correction are required. The trend toward ultrahigh fields (7 T) and the use of multichannel RF coils makes this ever more challenging. We investigated the constraints on reference phantoms, and implemented techniques for the absolute quantification of human liver phosphorus spectra acquired using a 10‐cm loop and a 16‐channel array at 7 T.

**Methods:**

The effect of phantom conductivity was assessed at 25.8 MHz (1.5 T), 49.9 MHz (3 T), and 120.3 MHz (7 T) by electromagnetic modeling. Radiofrequency field maps (${\text{B}}_{1}^{\pm }$) were measured in phosphate phantoms (18 mM and 40 mM) at 7 T. These maps were used to assess the correction of 4 phantom 3D‐CSI data sets using 3 techniques: phantom replacement, explicit normalization, and simplified normalization. In vivo liver spectra acquired with a 10‐cm loop were corrected with all 3 methods. Simplified normalization was applied to in vivo 16‐channel array data sets.

**Results:**

Simulations show that quantification errors of less than 3% are achievable using a uniform electrolyte phantom with a conductivity of 0.23‐0.86 S.m^−1^ at 1.5 T, 0.39‐0.58 S.m^−1^ at 3 T, and 0.34‐0.42 S.m^−1^ (16‐19 mM KH_2_PO_4(aq)_) at 7 T. The mean γ‐ATP concentration quantified in vivo at 7 T was 1.39 ± 0.30 mmol.L^−1^ to 1.71 ± 0.35 mmol.L^−1^ wet tissue for the 10‐cm loop and 1.88 ± 0.25 mmol.L^−1^ wet tissue for the array.

**Conclusion:**

It is essential to select a calibration phantom with appropriate conductivity for quantitative phosphorus spectroscopy at 7 T. Using an 18‐mM phosphate phantom and simplified normalization, human liver phosphate metabolite concentrations were successfully quantified at 7 T.

## 1 INTRODUCTION

Magnetic resonance spectroscopy is a useful tool for investigating in vivo metabolism, as it gives insight into the concentrations of various metabolites that are otherwise inaccessible using noninvasive methods.^1^ Metabolite concentrations often change in diseased tissue. Precise and accurate concentration measurements can differentiate different pathologies, stage the progress of disease, or monitor its treatment. For phosphorus (^31^P) MRS of the liver, disease targets include viral and alcoholic liver disease, cirrhosis, nonalcoholic fatty liver disease, and type 2 diabetes.^2^

Metabolite concentrations are calculated by normalizing the acquired signal and calibrating it to a reference of known concentration. The calibration reference can be either internal or external. An internal reference simplifies normalization, as several factors can be assumed to be the same for both the reference and the metabolite of interest. However, the concentration of an internal reference may not be known precisely. This necessitates either assuming a normal value for the reference concentration or reporting values as a ratio relative to the reference compound, such as the phosphocreatine/adenosine triphosphate (ATP) ratio, which is popular in cardiac ^31^P‐MRS. An external reference concentration can, however, be known precisely. In principle, this allows precise metabolite quantification that is independent of the operator, site, or scanner vendor, with all the advantages that brings.

Normalization often requires ${\text{B}}_{1}^{\pm }$ maps to be acquired from a phantom, particularly for non‐proton‐MRS. The implicit assumption is that the ${\text{B}}_{1}^{\pm }$ fields have the same spatial profile in the subject and in the phantom, but they may differ by a spatially independent factor due to effects such as coil loading. At low field strengths, this assumption is only expected to introduce small errors for phantoms of approximately in vivo concentrations.^3^, ^4^ Higher field strengths such as 7 T increase the ^31^P‐MRS SNR and improve metabolite quantification.^5^, ^6^ However, as field strength increases, the phantom’s dielectric properties become more important and the choice of phantom may be more tightly constrained.^7^

We therefore investigated the importance of phantom conductivity for absolute quantification of liver metabolites by electromagnetic field modeling and phantom experiments. Having identified a suitable phantom, we then assessed 3 possible approximations to the full normalization problem in phantoms, and applied these methods to report absolute metabolite concentrations in ^31^P‐MRS data sets acquired from the human liver using a 10‐cm‐loop RF coil. The most promising approach was further applied to 10 ^31^P‐MRS data sets acquired from the human liver using a 16‐element receive‐array RF coil.^6^

## 2 THEORY

MRS measurements can be used to determine the concentration [m] of a metabolite m. To do this, the signal at each voxel position ***r*** must be normalized by the scaling factor *n*_m_, as follows^8^:

(1) $$\left[\text{m}\right]/c=n_{\text{m}}\left(r\right)S_{\text{m}}\left(r\right),$$

where [m] is expressed in scanner‐specific units as [m]/*c*; and *c* is a calibration constant connecting to a known concentration reference standard. The normalization factor *n*_m_ consists of a partial‐saturation correction factor *F*_m_, which depends on the RF coil ${\text{B}}_{1}^{+}$, T_1_, and TR; a sensitivity correction factor *η,* which depends on ${\text{B}}_{1}^{-}$; and a volume correction factor.

With a receive array, each channel *k* gives a signal *S*_m,k_ from each voxel. Because these all arise from the same set of spins, Equation 1 applies for each channel, as follows:

(2) $$\left[\text{m}\right]/c=n_{\text{m},k}\left(r\right)S_{\text{m},k}\left(r\right)=n_{\text{m},j}\left(r\right)S_{\text{m},j}\left(r\right).$$

In practice, each channel’s signal also contains noise, which makes the per‐channel estimates of [m]/*c* imperfect. The estimate can be improved by taking a linear combination of the individual channels with some complex weighting factor ${\hat{w}}_{k}$, as follows:

(3) $$\left[\text{m}\right]/c=\sum_{k=1}^{N}{\hat{w}}_{k}\left(r\right)n_{\text{m},k}\left(r\right)S_{\text{m},k}\left(r\right).$$

To preserve the calibration *c*, the weights must be normalized as

(4) $${\hat{w}}_{k}=\frac{w_{k}}{\sum_{k=1}^{N}w_{k}}.$$

The best estimate of [m]/*c* is given by the weights that maximize the SNR of the combined spectrum.^9^ For example, the whitened singular value decomposition (WSVD) method^10^ gives the optimal uniform noise combination in most circumstances. For non‐proton‐MRS data, we previously showed that WSVD often gives a better SNR in the combined spectrum than using measured field maps.^11^

### 2.1 Phantom replacement

With a single receive element and a uniform phantom P, Equation 1 becomes

(5) $$\left[\text{P}\right]/c=n_{\text{P}}\left(r\right)S_{\text{P}}\left(r\right).$$

Dividing Equation 1 by Equation 5 cancels the factors of *c*, giving

(6) $$\frac{\left[\text{m}\right]}{\left[\text{P}\right]}=\frac{n_{\text{m}}\left(r\right)S_{\text{m}}\left(r\right)}{n_{\text{P}}\left(r\right)S_{\text{P}}\left(r\right)}.$$

If the acquisition protocols are identical, *n*_m_ = *n*_P_ and the concentration of the metabolite can be calculated from the concentration of the phantom multiplied by the ratio of the signals, as follows:

(7) $$\left[\text{m}\right]=\frac{S_{\text{m}}\left(r\right)}{S_{\text{P}}\left(r\right)}\left[\text{P}\right].$$

To extend this method to the combined signal, Equation 3 can be used as

(8) $$\frac{\left[\text{m}\right]}{\left[\text{P}\right]}=\frac{\sum_{k=1}^{N}{\hat{w}}_{\text{m},k}\left(r\right)n_{\text{m},k}\left(r\right)S_{\text{m},k}\left(r\right)}{\sum_{j=1}^{N}{\hat{w}}_{\text{P},j}\left(r\right)n_{\text{P},k}\left(r\right)S_{\text{P},k}\left(r\right)}.$$

However, as shown in the Supporting Information, if identical weights are used and Equation 7 holds, neither the signals nor the weights need to be normalized:

(9) $$\frac{\left[\text{m}\right]}{\left[\text{P}\right]}=\frac{\sum_{k=1}^{N}w_{k}\left(r\right)S_{\text{m},k}\left(r\right)}{\sum_{j=1}^{N}w_{j}\left(r\right)S_{\text{P},j}\left(r\right)}.$$

This allows [m] to be determined from the ratio of signals combined using any method.

If the T_1_ of the phantom and metabolite are different, the normalization factors are no longer equal. However, the only part of the normalization that depends on T_1_ is the saturation‐correction factor *F*_m_. If *F*_m_ is the same for each channel, it can be removed from the sum and applied after combination, as follows:

(10) $$\frac{\left[\text{m}\right]}{\left[\text{P}\right]}=\frac{F_{\text{m}}\left(r\right)\sum_{k=1}^{N}w_{k}S_{\text{m},k}\left(r\right)}{F_{\text{P}}\left(r\right)\sum_{j=1}^{N}w_{j}\left(r\right)S_{\text{P},j}\left(r\right)}.$$

### 2.2 Explicit normalization

For a CSI acquisition, normalization is complicated by the fact that the signal contributing to each voxel is an integral with the voxel point spread function (PSF),

(11) $$S_{\text{voxel}}\left(r\right)=\int S\left(s\right)\text{PSF}\left(r-s\right)\text{d}s,$$

where ***r*** is the position of each voxel and ***s*** is a position vector.

To account for this, the normalization constant *n*_m_ can be calculated as the inverse of the theoretical signal *I* in a uniform phantom of unit concentration,^12^ and Equation 3 can be rewritten as

(12) $$\left[\text{m}\right]/c=\sum_{k=1}^{N}{\hat{w}}_{k}\left(r\right)\frac{1}{I_{\text{m},k}\left(r\right)}S_{m,\text{k}}\left(r\right),$$

where

(13) $$I_{\text{m},k}\left(r\right)=\int \frac{B_{1,\text{k}}^{-}\left(s\right)\text{PSF}\left(r-s\right)}{F_{\text{m}}\left(s\right)}\text{d}s,$$

where *F*_m_ is saturation‐correction factor.

For a receive array, *F*_m_ is the same for each of the channels. It depends on TR, T_1_, and flip angle θ (i.e., the ${\text{B}}_{1}^{+}$). It differs from other metabolites in the same scan according to their T_1_ values. For a steady‐state acquisition, such as CSI, *F*_m_ at position ***r***is given by

(14) $$F_{\text{m}}\left(r\right)=\frac{1-\cos\theta \left(r\right)\;\cdot \;e^{-\frac{T_{\text{R}}}{T_{1,\text{m}}}}}{\sin\theta \left(r\right)\times \left(1-e^{-\frac{T_{\text{R}}}{T_{1,\text{m}}}}\right)}.$$

Both ${\text{B}}_{1}^{+}$ and ${\text{B}}_{1}^{-}$ can be determined from field maps acquired in phantoms.

### 2.3 Simplified normalization

If the variation of ${\text{B}}_{1}^{+}$ and ${\text{B}}_{1}^{-}$ across the region of space contributing to the main lobe of the PSF is relatively small, then one can approximate by using only the ${\text{B}}_{1}^{\pm }$ values at the central point of each voxel ***r***. Equation 13 can then be simplified to the following approximate form:

(15) $$I_{\text{m},k}=\frac{B_{1,k}^{-}\left(r\right)}{F_{\text{m}}\left(r\right)}\int \text{PSF}\left(r-s\right)\text{d}s=\frac{B_{1,k}^{-}\left(r\right)}{F_{\text{m}}\left(r\right)}V,$$

where *V* is determined using Equation 8 in Murphy‐Boesch et al^12^ as follows:

(16) $$V=\int \text{PSF}\left(r-s\right)\text{d}s.$$

This simplifies Equation 12 to

(17) $$\left[\text{m}\right]/c=\frac{F_{\text{m}}\left(r\right)}{V}\sum_{k=1}^{N}{\hat{w}}_{k}\left(r\right)\frac{1}{B_{1,k}^{-}\left(r\right)}S_{m,\text{k}}\left(r\right).$$

This is an attractively simple approximation, but we will show below that it is not always appropriate.

Although the sensitivity correction could be applied to each channel individually as the inverse of ${\text{B}}_{1}^{-}$, it can also be applied to the combined signal. If the weights are determined using the uniform noise Roemer formula, this combined sensitivity correction ***η ***can be determined by comparing the uniform noise and uniform sensitivity Roemer combination formulas (see Supporting Information)^9^ as follows:

(18) $$\eta \left(r\right)=\frac{1}{\sqrt{\hat{b}{\left(r\right)}^{†}{\left(\Psi^{-1}\right)}^{*}\hat{b}\left(r\right)}},$$

where $\hat{b}$ is the column vector of the receive field ${\text{B}}_{1}^{-}$ for each element; and Ψ is the noise covariance matrix (^† ^denotes the conjugate transpose and ^*^ denotes the conjugate).

Equation 15 becomes

(19) $$\left[\text{m}\right]/c=\frac{\eta \left(r\right)F_{\text{m}}\left(r\right)}{V}\sum_{k=1}^{N}w_{k}^{\text{RUN}}\left(r\right)S_{m,\text{k}}\left(r\right),$$

where $w_{k}^{\text{RUN}}$ are the Roemer uniform noise combination weights.

### 2.4 Calibration

The final step in absolute quantification is calibration. Because the calibration factor *c* does not change from scan to scan, the simplest method to determine it is to compare the normalized signal of the metabolite to that from a calibration reference standard R of known concentration [R]:

(20) $$c=\frac{\left[\text{R}\right]}{\sum_{k=1}^{N}w_{k}\left(r\right)n_{\text{R},k}\left(r\right)S_{\text{R},k}\left(r\right)},$$

where *n*_R_ can be determined according to either Equation 12 or Equation 19. As long as the reference signal is normalized in the same way as the target metabolite, it could be internal or external. The benefits of an external reference are that its concentration can be known precisely. The estimate of *c* can be improved by averaging across multiple voxels.

The requirements for implementing Equations 10, 12, and 19 are detailed in Table 1.

**Table 1:** Implementation of correction methods

Method	Correction	Weights	Assumptions
Method 1: Phantom replacement	Equation 10: $\frac{\left[\text{m}\right]}{\left[\text{P}\right]}=\frac{F_{\text{m}}\left(r\right)\sum_{k=1}^{N}w_{k}S_{\text{m},k}\left(r\right)}{F_{\text{P}}\left(r\right)\sum_{j=1}^{N}w_{j}\left(r\right)S_{\text{P},j}\left(r\right)}$ and saturation correction per Equation 14: $F_{\text{m}}\left(r\right)=\frac{1-\cos\theta \left(r\right)\;\cdot \;e^{-\frac{T_{\text{R}}}{T_{1,\text{m}}}}}{\sin\theta \left(r\right)\;\times \;\left(1-e^{-\frac{T_{\text{R}}}{T_{1,\text{m}}}}\right)}$	WSVD applied to the phantom. The same weights are applied to the in vivo data.	All parameters except saturation correction are identical in both scans. Rather than scanning multiple phantoms with different T_1_ values, both phantom and metabolite signals were saturation‐corrected using Equation 14.
Method 2: Explicit normalization	Equation 12: $\left[\text{m}\right]/c=\sum_{k=1}^{N}{\hat{w}}_{k}\left(r\right)\frac{1}{I_{\text{m},k}\left(r\right)}S_{\text{m},k}\left(r\right)$	WSVD applied separately to in vivo and phantom data. Weights normalized to sum to 1, according to Equation 4.	Assumes uniform metabolite concentration across the PSF.
Method 3: Simplified normalization	Equation 19: $\left[\text{m}\right]/c=\frac{\eta \left(r\right)F_{\text{m}}\left(r\right)}{V}\sum_{k=1}^{N}w_{k}^{\text{RUN}}\left(r\right)S_{\text{m},k}\left(r\right)$	WSVD applied separately to in vivo and phantom data.	Assumes the ${\text{B}}_{1}^{\pm }$ values at the central point of each voxel can be used for normalization (i.e., either PSF is sharply peaked and/or ${\text{B}}_{1}^{\pm }$ are spatially slowly varying).

Abbreviations: PSF, point spread function; WSVD, whitened singular value decomposition.

## 3 METHODS

### 3.1 Electromagnetic simulations

CST Studio Suite 2016 (CST AG, Darmstadt, Germany) was used to simulate ${\text{B}}_{1}^{+}$ fields of a 10‐cm‐loop coil centered above various phantoms (described below) and 2 human voxel models: Laura and Gustav. These models are representative in that Laura is a slim woman and Gustav is a well‐built man. For the human voxel models, simulations used values of conductivity and permittivity provided in CST Studio Suite 2016 for each tissue type. For each phantom, we used calibrated conductivity values and fixed the permittivity value equal to the relative permittivity of water ($\epsilon_{r}\;=\;79$), as the permittivity of ionic solutions varies very little at concentrations less than 50 mM.^13^, ^14^, ^15^ In each case, the fields were simulated at 25.9, 49.9, and 120.3 MHz. The coil was tuned to each frequency and matched to 50 Ω on the phantom. The ${\text{B}}_{1}^{+}$ field was sampled along a perpendicular line through the center of the coil, passing through the phantom. The ${\text{B}}_{1}^{+}$ profiles were normalized to a “reference fiducial” 10 mm behind the face of the coil, and then a ratio was taken and compared with an average from the Laura and Gustav models, to assess the extent to which conductivity and permittivity effects altered the shape of the ${\text{B}}_{1}^{+}$ profile in the phantoms compared with the human voxel models. Finally, the mean and SD of the ratio values across the depth of the liver were calculated to give a single bias, SD, and RMS error (RMSE) for each simulation.

First, simulations were performed to determine the minimum size of the phantom, to avoid boundary effects (Supporting Information Figures S1 and S2). Based on the size simulations, a jerry can phantom of dimensions 280 × 280 × 450 mm^3^ was selected. A CST model of this phantom was made, and 11 conductivities between 0.02 S.m^−1^ and 2 S.m^−1^ were simulated.

### 3.2 Acquisition of field maps

Two phosphate (K_2_HPO_4[aq]_) phantoms were made up in jerry cans, with concentrations 18 mM (0.36 S.m^−1^) and 40 mM (0.89 S.m^−1^) (Supporting Information Table S1).

Data were acquired on a whole‐body Magnetom 7T system (Siemens, Erlangen, Germany) using a 10‐cm ^31^P loop. Coil location and loading were calculated using a phenylphosphonic acid fiducial.^5^ The T_1_ for each phantom was determined using nonlocalized inversion‐recovery FID signals. Ten ^31^P 3D gradient‐recalled echo images (32 × 16 × 20, 15.6 × 15.6 × 15.6 mm^3^, 100‐ms TR, total time per image: 1 hour 20 minutes) were acquired with transmit voltages varying between 2 V_RMS_ and 270 V_RMS_. Field maps were calculated using MATLAB’s lsqcurvefit to fit the intensities acquired at various voltages to Equation 11, as follows:

(21) $$I\left(V\right)=\frac{a\sin bV\times \left(1-e^{-\frac{T_{R}}{T_{1}}}\right)}{1-\cos bV\;\cdot \;e^{-\frac{T_{R}}{T_{1}}}},$$

where constant *a* is proportional to the receive sensitivity, and *b* is the number of degrees per volt at that pixel (i.e., the transmit field map). The maps are then converted to ${\text{B}}_{1}^{+}$ in hertz per volt by multiplication of the flip angle per hertz calculated for the excitation pulse used in the gradient‐recalled echo acquisition.

The field maps can be applied to any arbitrary position of the coil by linear interpolation. With knowledge of the pulse in a given scan, the flip angle can be calculated for any voxel.

The field map acquisition scans were repeated for a 16‐channel receive array (Rapid Biomedical, Rimpar, Germany), consisting of a single 28 × 27 cm^2^ transmit loop and a 4 × 4 matrix of 8 × 5.5 cm^2^ diameter flexible receive loops.^16^

### 3.3 Phantom validation of field‐map methods

To calibrate and test the sensitivity maps for the 18‐mM phantom, 2 CSI scans were performed for each of the coils (i.e., the 10‐cm loop and the 16‐channel receive array).

In the “calibration” scan, a 1‐second TR, 10 average acquisition‐weighted UTE‐CSI sequence was used to acquire a 16 × 16 × 8 matrix of spectra over a 270 × 240 × 200 mm^3^ FOV during the same session as the gradient‐recalled echo images.^17^ The total time required for the acquisition of the field maps and data required for calibration was 20 hours. In a separate (“repeat”) scan, a further 16 × 16 × 8 CSI matrix was acquired on the 18‐mM phantom in 28 minutes, using the same acquisition parameters but without gradient‐recalled echo field mapping.

Three methods were used to process the data (as indicated in Table 1):

**Method 1:** Phantom replacement. Each spectrum was analyzed using the OXSA toolbox.^18^ The fitted amplitudes were corrected for saturation using the ${\text{B}}_{1}^{+}$ at the central point of each voxel to calculate the flip angle, and then applying Equation 14. The corrected amplitudes were then divided by the corrected amplitude from the calibration scan, accounting for relative position and orientation of the coil and the coil loading (i.e., applying Equation 10).

**Method 2:** Explicit normalization. Each spectrum was analyzed using the OXSA toolbox.^18^ The received signal was normalized to the expected signal across the PSF using the phantom ${\text{B}}_{1}^{\pm }$ (Equation 12).

**Method 3:** Simplified normalization. Each spectrum was analyzed using the OXSA toolbox.^18^ The fitted amplitudes were corrected using the ${\text{B}}_{1}^{+}$ at the central point of each voxel to determine *F* (using Equation 14), the ${\text{B}}_{1}^{-}$ at the central point of each voxel to determine *η* (using Equation 18), and then applying Equation 17. Volume correction was based on the 50% FWHM of the PSF, rather than the full PSF.

The same methods were used for the 16‐channel receive array. Before analysis, the single‐channel spectra were combined using WSVD. For the phantom replacement method (Method 1), WSVD was performed on the calibration CSI scan, and in each of the following scans the same weights were used for coil combination. For the explicit normalization (Method 2), the WSVD weights are normalized according to Equation 4 such that Σ|*w*| = 1.

A calibration factor was then calculated by dividing the true concentration of each phantom by the mode of the corrected amplitudes (see Equation 20).

The mean and the SDs of each of the field maps and concentration maps were calculated from the 5th to 95th percentile data. The bias of the concentration maps was calculated as the deviation of the mean from the 18 mM or 40 mM value. The RMSE was calculated as the square root of the bias squared plus the SD squared.

### 3.4 In vivo assessment with a 10‐cm‐loop coil

Four subjects (3 male and 1 female, 26 ± 4 years, body mass index [BMI] 20.8 ± 3.2 kg.m^‐2^) were scanned after an overnight fast, in accordance with procedures approved by our local ethics committee. UTE‐CSI spectra from within the liver were acquired and analyzed as previously described^6^ using a 10‐cm ^31^P loop for the main acquisition. In short, a 1‐second TR, 10‐average acquisition‐weighted UTE‐CSI sequence was used to acquire a 16 × 16 × 8 matrix of liver spectra over a 270 × 240 × 200 mm^3^ FOV in a total acquisition duration of 28 minutes. Overlying skeletal muscle was suppressed using a BISTRO saturation band.^19^ The spectra were then automatically processed so that only the good‐quality voxels within the liver (from all slices covering the liver) remained for the final determination of concentrations.^6^ Each spectrum was analyzed using fitting from the OXSA toolbox with constrained Voigt lineshapes,^18^ and saturation‐corrected and sensitivity‐corrected using field maps acquired using the 18‐mM phosphate phantom according to the 3 methods described previously. Signal from the spherical phenylphosphonic acid fiducial, built into the housing on the rear of the coil, was used to determine the actual flip angle in each voxel, which was then used for saturation correction. Cramér‐Rao lower‐bound (CRLB) measures of error^20^ were calculated from the analysis of each spectrum and combined with the error arising from the normalization and calibration. The intrasubject SD was calculated as the SD of all the voxels used in the determination of the concentration.

### 3.5 In vivo assessment with a 16‐element receive array

Data from 10 volunteers (6 male and 4 female, 27 ± 5 years, BMI 22.5 ± 1.5 kg.m^‐2^) that were acquired for a recent study after an overnight fast, in accordance with guidelines from our local ethics committee, were retrospectively analyzed.^6^ In the original study, metabolite concentrations were computed assuming a γ‐ATP concentration of 2.65 mmol.L^−1^ wet tissue (as an endogenous reference). Previously described automated quality assurance tests^6^ were used to define all good‐quality liver voxels (based on SNR and phosphocreatine contamination) for further analysis. Each spectrum was then fitted with the OXSA toolbox and constrained Voigt lineshapes,^18^ and saturation‐corrected and sensitivity‐corrected using B_1_ at the central point of each voxel (Method 3) to determine metabolite concentrations using exogenous reference. The CRLBs were calculated from the analysis of each spectrum and combined with the error arising from the normalization and calibration. The intrasubject SD was calculated as the SD of all voxels used in the determination of the concentration. Mean values were compared with previously reported literature values using Welch’s t‐test.

## 4 RESULTS

### 4.1 Electromagnetic modeling

The ${\text{B}}_{1}^{+}$ values in the liver of the human voxel models were normalized to the ${\text{B}}_{1}^{+}$ at the simulated fiducial, to account for loading. Normalized ${\text{B}}_{1}^{+}$ values differed between the Gustav and Laura models by 0.7% at 1.5 T, 1.6% at 3 T, and 7.0% at 7 T. Matching the phantom conductivity to liver tissue conductivity of 0.5 S.m^−1^ gives a 1.5% error at 3 T and 10% error at 7 T, but it is possible to more closely match the in vivo values with slightly lower conductivities.

**Figure 1:**
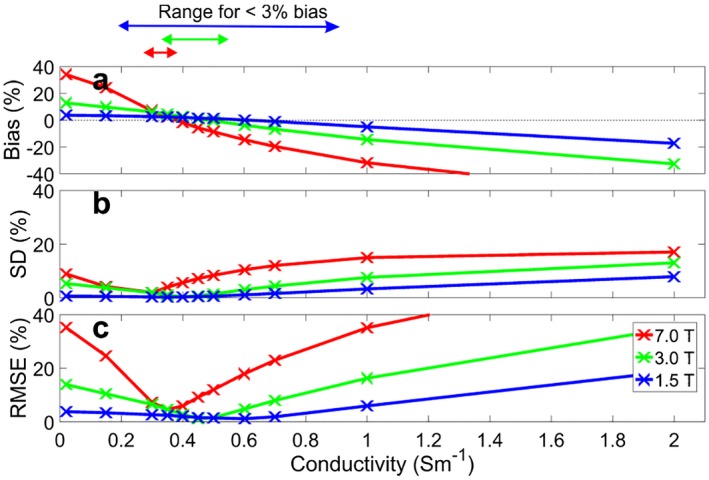
${\text{B}}_{1}^{+}$ error for uniform phantoms of conductivity (0.02‐2 S.m^−1^) versus human liver models. A, Bias relative to the simulated human liver voxel models. B, Standard deviation. C, Root mean square error (RMSE)

The variation in ${\text{B}}_{1}^{+}$ due to difference in conductivity and field strength is shown in Figure 1. The conductivities required for certain levels of error are summarized in Table 2.

**Table 2:** Conductivities required at different field strengths, according to CST simulations

Field strength	Conductivity for <3% bias (S.m^−1^)	Conductivity for <0.5% bias (S.m^−1^)	Bias crossing point (S.m^−1^)	Conductivity required to minimize RMSE (S.m^−1^)
1.5	0.23‐0.86	0.57‐0.66	0.62	0.6
3	0.39‐0.58	0.47‐0.50	0.48	0.45
7	0.34‐0.42	0.37‐0.38	0.38	0.35

### 4.2 Acquisition and validation of field maps

**Figure 2:**
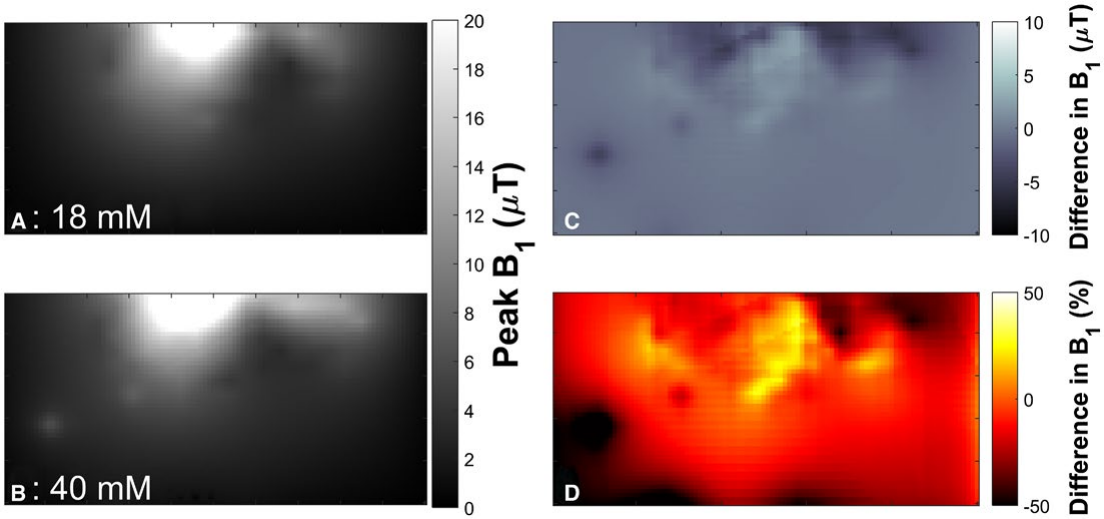
The 10‐cm‐loop phantom field maps. The ${\text{B}}_{1}^{+}$ field maps are sampled at a 4 × 4.5 mm^2^ resolution over a 400 × 200 mm^2^ FOV. A, The ${\text{B}}_{1}^{+}$ field map from the 18‐mM phantom. B, Field map from the 40‐mM phantom. C, Difference between the 2 maps in microteslas ([A]–[B]). D, Difference as a percentage

The T_1_ mean ± fitting CRLB for the 18‐mM phosphate phantom was 15.9 ± 1.2 seconds, and the value for the 40‐mM phosphate phantom was 7.8 ± 1.7 seconds. Figure 2 shows the ${\text{B}}_{1}^{+}$ field maps for both the 18‐mM and 40‐mM phantoms, as well as the difference between the 2 maps.

### 4.3 Application of field maps to phantom data

**Figure 3:**
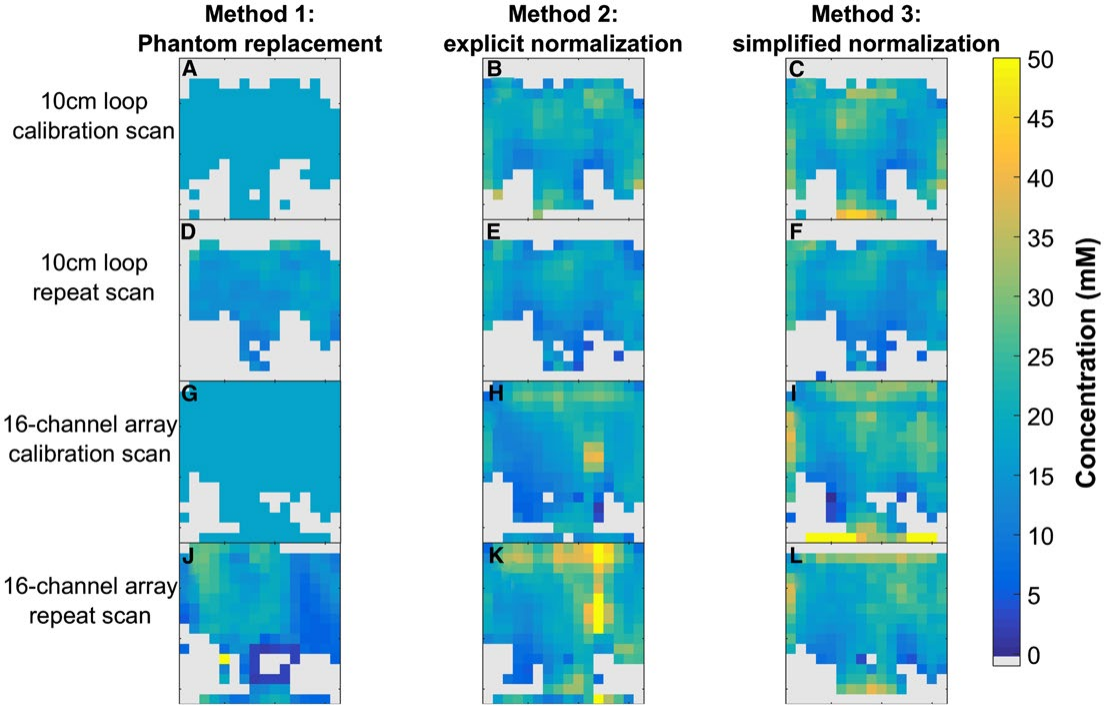
Midtransverse slice illustrates the 3D data acquired from four 16 × 16 × 8 UTE‐CSI acquisitions of an 18‐mM K_2_HPO_4_ phantom, with sensitivity correction performed using 3 different methods. A‐F, The 10‐cm‐loop data. G‐I, The 16‐channel array data. A‐C,G‐I, Calibration scans. D‐F,J‐I, Repeat scans

**Figure 4:**
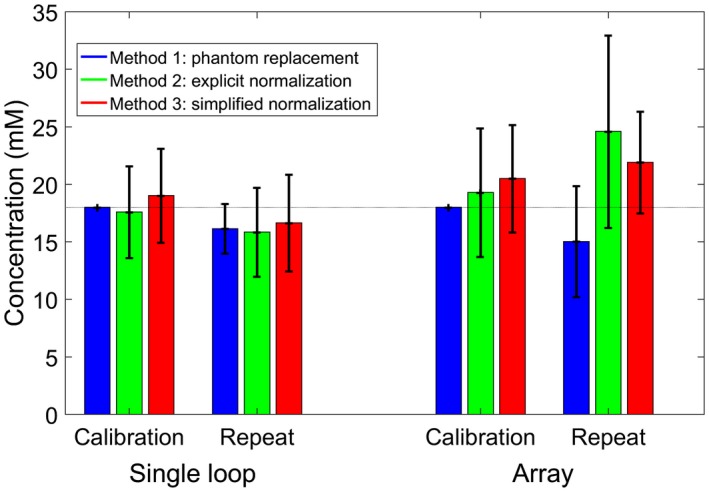
Mean ± SD values from correction of UTE‐CSI acquisitions of an 18‐mM K_2_HPO_4_ phantom. The bias, SD, and RMSE values are given in Table 3. Data are taken from all voxels inside the phantom in all relevant slices

The concentration maps corrected by each of the 3 correction methods are shown in Figure 3 for the 10‐cm loop and the 16‐channel array. The bias, SD, and RMSE for each of the correction methods in the calibration and repeat scans is given for both the 10‐cm loop and the 16‐channel receive array in Table 3 and shown in Figure 4. The bias in the calibration scans arises from the difference between the mode and the mean of the corrected amplitudes. As the phantom replacement data are defined relative to the calibration scans, the bias, SD, and RMSE are all zero for those scans.

**Table 3:** Error data from correction of UTE‐CSI acquisitions of an 18‐mM K_2_HPO_4_ phantom

	Bias (mM)	SD (mM)	RMSE (mM)
**10‐cm‐loop coil**			
Method 1: Phantom replacement			
Calibration scan	0 by definition		
Repeat scan	−1.85	2.16	2.85
Method 2: Explicit normalization			
Calibration scan	−0.41	3.98	4.00
Repeat scan	−2.15	3.87	4.43
Method 3: Simplified normalization			
Calibration scan	1.02	4.07	4.20
Repeat scan	−1.35	4.20	4.41
**16‐channel receive array**			
Method 1: Phantom replacement			
Calibration scan	0 by definition		
Repeat scan	−2.97	4.84	5.68
Method 2: Explicit normalization			
Calibration scan	1.29	5.58	5.73
Repeat scan	6.59	8.37	10.65
Method 3: Simplified normalization			
Calibration scan	2.51	4.66	5.29
Repeat scan	3.91	4.43	5.91

Note

The mean and SD are plotted in Figure 4.

The RMSE for the array is larger than for the 10‐cm loop (5.29‐10.65 mM versus 2.85‐4.43 mM). Simplified normalization (Method 3) gives similar RMSE to the full explicit normalization (Method 2) for the 10‐cm loop (both approximately 4‐4.4 mM), but smaller errors for the array (5.29‐5.91 mM versus 5.73‐10.65 mM).

### 4.4 Application to 10‐cm‐loop in vivo data

The average concentrations for γ‐ATP acquired with the 10‐cm loop and corrected by each of the 3 methods are given in Table 4, with the mean CRLB and both intrasubject and intersubject SD.

**Table 4:** In vivo hepatic γ‐ATP concentrations and errors acquired using a 10‐cm loop

Method	γ‐ATP intersubject mean ± SD (mmol.L^−1^ wet tissue)	γ‐ATP mean CRLB (mmol.L^−1^ wet tissue)	γ‐ATP intrasubject SD (mmol.L^−1^ wet tissue)
Method 1: Phantom replacement	1.39 ± 0.30	0.12 ± 0.02	0.66 ± 0.21
Method 2: Explicit normalization	1.73 ± 0.35	0.13 ± 0.02	0.57 ± 0.16
Method 3: Simplified normalization	1.46 ± 0.23	0.43 ± 0.07	0.56 ± 0.06

Note

The concentrations are the mean from all subjects. The fitting Cramér‐Rao lower‐bound (CRLB) and intrasubject SD are given as a mean ± SD across all subjects.

The mean CRLBs include the lowest bounds on all of the errors due to the analysis, normalization, and calibration of the signal. Using simplified normalization (Method 3), the intrasubject SDs are not significantly different from the CRLBs (*P* > 0.1). This implies that the errors in the final concentrations are caused by a lack of precision in the field maps, as low accuracy in the field maps would increase the SD in vivo. Voxel overlap was not accounted for when calculating the intrasubject mean and SD.

### 4.5 Application to 16‐channel receive‐array in vivo data

**Figure 5:**
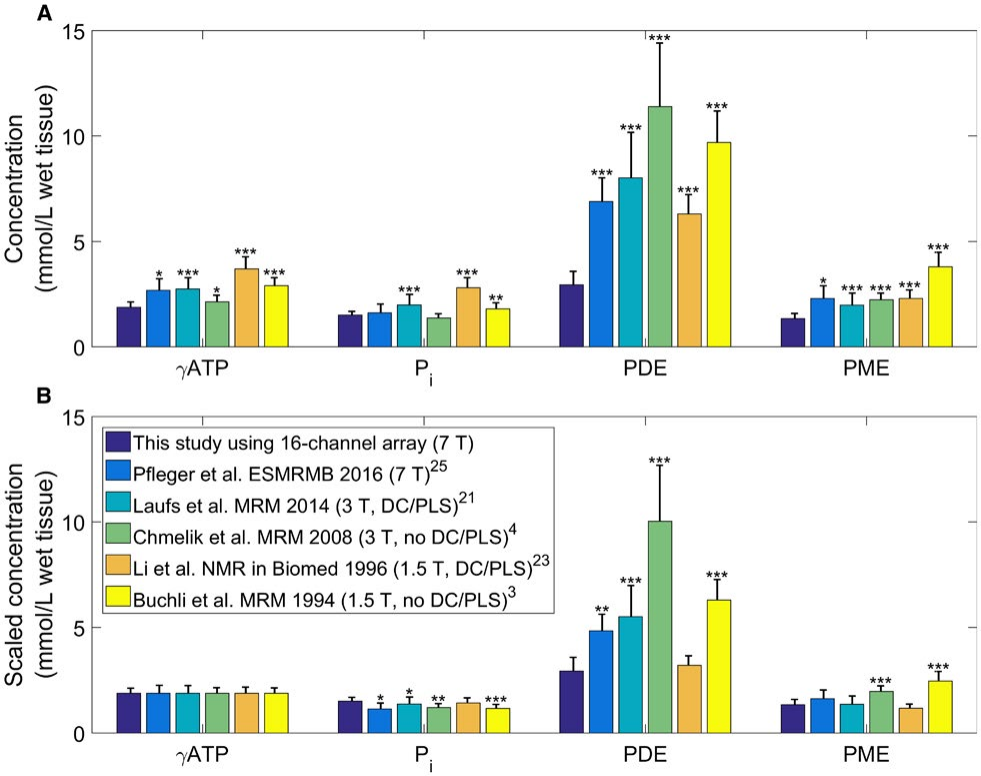
Comparison of normal‐liver phosphorus (^31^P) metabolite concentrations from this study against the literature.^3^, ^4^, ^23^, ^25^, ^27^ A, Reported concentrations. B, Concentrations scaled so that each gamma adenosine triphosphate (γ‐ATP) value matches the value reported in this study (1.88‐mmol.L^−1^ wet tissue). The SDs are shown by the error bars on each bar. Stars indicate the level of significance of the difference from this study: ^*^ *P* < 0.05, ^**^ *P* < 0.01, ^***^ *P* < 0.001. The legend gives the studies with their field strength, and whether they use decoupling and phospholipid saturation. Abbreviations: DC, decoupling; PDE, phosphodiester; P_i_, inorganic phosphate; PLS, phospholipid saturation; PME, phosphomonoester

The average concentrations for each of the visible metabolites acquired with the 16‐channel receive array and using the simplified normalization (Method 3) are given in Table 5. The concentrations of γ‐ATP, P_i_, the summed phosphomonoester (PME) peaks, and the summed phosphodiester (PDE) peaks are compared with literature values in Figure 5. Figure 5A shows the reported results, whereas Figure 5B shows the results scaled to use our average 1.88‐mmol.L^−1^ wet tissue value for γ‐ATP. Sample spectra are shown in Supporting Information Figure S3.

**Table 5:** In vivo hepatic ^31^P metabolite concentrations and errors acquired using a 16‐channel receive array and normalized using the simplified method (Method 3)

Metabolite	Mean ± intersubject SD (mmol.L^−1^ wet tissue)	Mean CRLB (mmol.L^−1^ wet tissue)	Mean intrasubject SD (CoV) (mmol.L^−1^ wet tissue)
α‐ATP	1.80 ± 0.25	0.83 ± 0.16	0.99 (54.9%) ± 0.31
γ‐ATP	1.88 ± 0.25	0.86 ± 0.17	0.96 (51.2%) ± 0.33
P_i_	1.50 ± 0.20	0.66 ± 0.12	0.59 (39.1%) ± 0.18
GPC	1.59 ± 0.36	0.66 ± 0.15	0.55 (34.7%) ± 0.14
GPE	1.35 ± 0.31	0.57 ± 0.13	0.52 (38.4%) ± 0.15
PC	0.69 ± 0.16	0.30 ± 0.07	0.22 (31.1%) ± 0.04
PE	0.65 ± 0.12	0.28 ± 0.05	0.24 (37.1%) ± 0.06
NAD^+^	1.95 ± 0.28	1.09 ± 0.19	1.29 (66.0%) ± 0.38
UDPG	1.25 ± 0.17	1.00 ± 0.16	0.97 (77.5%) ± 0.21
PtdC/PEP	0.73 ± 0.17	0.43 ± 0.08	0.52 (71.4%) ± 0.24

Note

The concentrations are the mean from all subjects. The fitting CRLB and intrasubject SD are given as a mean ± SD across all subjects.

Abbreviations: CoV, coefficient of variation; GPC, glycerophosphorylcholine; GPE, glycerophosphorylethanolamine; NAD, nicotinamide adenine dinucleotide; PC, phosphocholine; PE, phosphoethanolamine; PEP, phosphoenolpyruvate; P_i_, inorganic phosphate; PtdC, phosphatidylcholine; UDPG, uridine diphosphate glucose.

## 5 DISCUSSION

A simple phosphate phantom was used to calculate concentrations from human in vivo hepatic ^31^P‐MRS data. However, care was required to choose the conductivity of the phantom such that the B_1_ profile of the phantom approximately matched the B_1_ profile in vivo at 7 T.

The conductivity of liver tissue increases from 0.38 S.m^−1^ at 25.8 MHz (1.5 T for ^31^P) to approximately 0.5 S.m^−1^ at 120.3 MHz (7 T for ^31^P).^21^, ^22^ In contrast, the optimal conductivities, based on a comparison with the average ${\text{B}}_{1}^{\pm }$ fields from the Gustav and Laura models, for measuring in vivo liver spectra decreases from 0.6 S.m^−1^ at 1.5 T to 0.35 S.m^−1^ at 7 T. These values differ from the liver conductivities because the liver is surrounded by fat and muscle, affecting the average ${\text{B}}_{1}^{\pm }$ fields. The overall effect of the multiple tissues depends on the field strength. For each field strength, both the bias and the SD are minimal at around the same conductivities. The small discrepancies can be accounted for by using the RMSE to minimize the total error.

The complexity of the in vivo models meant that the difference between the Gustav and Laura models begins to have an effect at 7 T, with a 7% difference compared with the 1.6% at 3 T and 0.7% at 1.5 T.

The simulations were done for ^31^P resonant frequency at 1.5 T, 3 T, and 7 T (i.e., 25.8 MHz, 49.9 MHz, and 120.3 MHz). These frequencies are approximately equivalent to 0.5 T, 1 T, and 3 T for proton (^1^H), or 2.3 T, 4.5 T, and 11 T for carbon‐13 or sodium‐23. The phantom replacement methods described in this paper are not used commonly for ^1^H, as the increased SNR for ^1^H allows direct in vivo measurement of B_1_. The methods are used for carbon and sodium, but the issues at each field strength are reduced due to the lower gyromagnetic ratio.

The difference in the field maps between the 18‐mM and 40‐mM phantoms (0.36 S.m^−1^ and 0.89 S.m^−1^) are clearly visible in Figure 2. The individual maps show the “twisting” that is expected of a B_1_ field in a conductive material at ultrahigh field.^7^ The difference maps show that the error can be over 50% of the mean value. This can lead to significant errors in calculated metabolite concentrations if the wrong field map is applied (i.e., a field map acquired using a phantom with conductivity outside the simulated acceptable range).

If the corrections were perfect, the SD of concentrations in the phosphate phantom should reflect only spatially uniform thermal noise in the measurement. However, after both saturation and sensitivity correction are applied to the amplitudes of the phosphate phantom, there was residual structure, and the SD was 12%‐22% for the 10‐cm loop and 25%‐47% for the array (depending on the method used). This correction includes errors from fitting the T_1_ values, any artifacts in the image, fitting the voltage curve, and interpolation onto the CSI grid. These are the smallest SDs that can be expected to be included from the absolute quantification of in vivo values.

Applying the field map to a CSI scan acquired in a separate session does not appear to increase the SDs, but gives 0.3‐mM to 5.2‐mM additional bias. This is likely due to small changes in the ${\text{B}}_{1}^{\pm }$ field, and due to errors in the transformation from 1 coil position to another. The additional bias is negative for the 10‐cm‐loop methods and the phantom replacement method for the array, but positive for methods 2 and 3 for the array. If a similar bias is seen in vivo, then the γ‐ATP concentrations reported for the 10‐cm loop may be underestimated by about 10%, and those reported for the array overestimated by up to 20%.

The mean CRLBs include the lowest bounds on all of the errors due to the analysis, normalization, and calibration of the signal. The intrasubject SDs are not significantly different from the CRLBs (*P* > 0.1). This implies that the errors in the final concentrations are caused by a lack of precision in the field maps, as low accuracy in the field maps would increase the SD in vivo.

There are potentially several ways to reduce the error on the final concentrations that are introduced due to B_1_. For example, a more complex phantom may be used to more precisely match the in vivo B_1_ field, such as by optimizing permittivity as well as conductivity, or more time could be taken to acquire higher SNR data used for both the ${\text{B}}_{1}^{\pm }$ maps and the calibration factor. However, the reduction in error for each individual improvement is marginal (i.e., 4 hours of additional time spent per voltage or 40 hours for the whole acquisition), and to double the SNR would only improve the error by 2%, according to Monte Carlo simulations (not shown). Similarly, acquiring ${\text{B}}_{1}^{\pm }$ data with higher resolution may improve precision by reducing the ${\text{B}}_{1}^{\pm }$ range within a voxel. However, this would take significantly longer to acquire. The population included in this study, recruited for a previous study,^6^ had a relatively narrow BMI range, which could affect the measured intersubject ${\text{B}}_{1}^{\pm }$ distribution. However, all simulations were performed on both the Gustav and Laura voxel models, thus accounting for the typical range of BMI among the subjects we expect to scan at 7 T.

The γ‐ATP in vivo concentrations calculated using both the data acquired using the 10‐cm loop (1.39‐1.73 mmol.L^−1^ wet tissue) and the 16‐channel array (1.88‐mmol.L^−1^ wet tissue) are low compared with literature values of 1.9 mmol.L^−1^ to 3.7 mmol.L^−1^ wet tissue.^3^, ^4^, ^23^, ^24^, ^25^, ^26^ Figure 5 compares our values to literature values at 1.5 T, 3 T and 7 T, both with and without decoupling and phospholipid saturation.^3^, ^4^, ^23^, ^25^, ^27^ Acquiring spectra at 1.5 T and 3 T without phospholipid saturation and decoupling increases the measured values for PME and PDE due to the underlying endoplasmic reticulum signal.^28^, ^29^ The phosphatidylcholine signal also contributes to the PME peak at low field strengths.^30^ Unlike ATP, in vitro PDE peak concentrations are assumed to be consistent with in vivo values.^31^ Converting published mmol.kg^−1^ wet weight values using 1.054 kg.L^−1^ specific gravity,^32^ glycerophosphorylethanolamine has an in vitro concentration of 2.59 ± 0.39 mmol.L^−1^ wet tissue and glycerophosphorylcholine has a concentration of 2.48 ± 0.48 mmol.L^−1^ wet tissue.^31^ The combined PDE concentration is significantly lower than the literature values (*P* < 0.01),^3^, ^4^, ^23^, ^25^, ^27^ but significantly higher than the values reported in this study (*P* = 0.01, glycerophosphorylcholine 1.59 ± 0.36 mmol.L^−1^ wet tissue, glycerophosphorylethanolamine 1.35 ± 0.31 mmol.L^−1^ wet tissue). This difference is seen even for the studies in which decoupling and phospholipid suppression are expected to make accurate quantification of the PDE possible.^23^, ^25^, ^27^ The challenge in determining the optimal method arises due to the unknown concentration of ^31^P metabolites that are visible using MR techniques. Several studies have shown that some proportion of ATP, adenosine diphosphate, and inorganic phosphate are MR‐invisible.^33^, ^34^, ^35^, ^36^, ^37^, ^38^ It is likely that the same is true for the PDE and PME peaks. This may contribute to the difference between our reported values for PDE and the higher values measured in vitro.

As the literature concentrations have small SDs, the results are expected to be reproducible but biased. This bias is introduced during the calibration step. For example, Pfleger et al calibrated their results using a 0.65‐S.m^−1^ phantom at 7 T,^27^ and Laufs et al calibrated their results using a 50‐mM K_2_HPO_4_ phantom at 3 T (approximately 0.89 S.m^−1^).^23^ Both of these phantoms are expected to introduce about 20% bias when used for sensitivity correction of in vivo liver spectra (Figure 1).

In this study and the work of Pfleger et al^27^ and Laufs et al,^23^ the calibration phantom’s *conductivity* was optimized to mimic RF field effects similar to the human body. However, so far, we have neglected the much higher permittivity in our phantom (about 80)^13^, ^14^, ^15^ compared with the permittivity in human tissue at 120.3 MHz (e.g., about 65 for liver or muscle and about 6 for fat).^39^ The remaining bias and mismatch from our phantom replacement procedure might be improved by optimizing both the phantom conductivity *and* the phantom permittivity, which is more complex to realize but shown to be feasible.^40^

Nevertheless, even a biased “absolute” quantification remains useful. This is because the results are reproducible, so these methods give additional information over relative quantification (i.e., metabolite ratios), such as in longitudinal studies of the same subjects. Furthermore, we cannot be sure that all sources of biological variability in liver tissue metabolites have been identified so far. Therefore, it is key that every center must obtain their own healthy volunteer values under the same experimental conditions as their future planned studies, rather than relying on literature concentrations at this time.

## 6 CONCLUSIONS

Using simulations, we have determined that the acceptable range of conductivities for a uniform phosphate phantom is reduced at 7 T (0.34‐0.42 S.m^−1^) compared with 3 T (0.39‐0.58 S.m^−1^) and 1.5 T (0.23‐0.86 S.m^−1^). A 0.35‐S.m^−1^ uniform phosphate phantom (18 mM K_2_HPO_4[aq]_) best matches in vivo ${\text{B}}_{1}^{+}$ for human liver ^31^P‐MRS at 7 T. This phantom was used to normalize and calibrate in vivo ^31^P‐MRS of the human liver. Full explicit normalization (Method 2) improves SDs by 1%‐2% compared with simplified normalization (Method 3) using a single loop coil. However, Method 3 provides the smallest SDs for the receive array. If care is taken with the selection of phantoms and correction methods, absolute quantification is possible using a 16‐channel array at 7 T.

## Supporting information


**FIGURE S1** The B_1_ error for different depth cylindrical phantoms
**FIGURE S2** The B_1_ error for different width cylindrical phantoms
**FIGURE S3** Sample spectra acquired from the human liver in a healthy volunteer using the 16‐element array coil. A, Transverse localizer image overlaid with the saturation band (yellow) and CSI matrix (red). Voxels from this slice that met the quality criteria and were used for further analysis are highlighted. (Note that all high‐quality liver voxels from all slices were used in the analysis.) B‐D, Representative spectra from the corresponding voxels highlighted in (A). B, Skeletal muscle showing phosphocreatine signal. C,D, Liver showing negligible phosphocreatine
