# Supplementary material for: Feasibility of absolute quantification for 31P MRS at 7 T
Source: Magn Reson Med. 2019 Mar 20;82(1):49–61. doi: 10.1002/mrm.27729 (PMC6492160; doi:10.1002/mrm.27729)
Supplement: Supplementary file 1 — FIGURE S1 The B1 error for different depth cylindrical phantoms FIGURE S2 The B1 error for different width cylindrical phantoms FIGURE S3 Sample spectra acquired from the human liver in a healthy volunteer using the 16‐element array coil. A, Transverse localizer image overlaid with the saturation band (yellow) and CSI matrix (red). Voxels from this slice that met the quality criteria and were used for further analysis are highlighted. (Note that all high‐quality liver voxels from all slices were used in the analysis.) B‐D, Representative spectra from the corresponding voxels highlighted in (A). B, Skeletal muscle showing phosphocreatine signal. C,D, Liver showing negligible phosphocreatine [file MRM-82-49-s001.docx]

# Supporting information for “The feasibility of absolute quantification for ^31^P magnetic resonance spectroscopy at 7T”

## Phantom replacement for receive arrays

Consider the case where the signals from *N* individual elements are combined with arbitrary, but identical, weights, and a ratio of a metabolite scan m and a phantom scan P is taken.

$\frac{\sum_{k=1}^{N} w_{k}S_{m,k}}{\sum_{j=1}^{N} w_{j}S_{P,j}}$ [S1]

Where *w_k/j_* are the weights of the *k^th^*/*j*^th^ channel, and *S* is the signal.

Rearranging Eq. 7 to give *S*_P_ in terms of *S*_m_:

$S_{P}=\frac{\left[ P \right]}{[m]} S_{m}$. [S2]

where [P] and [m] are the concentrations of the phantom and metabolite. This can be substituted into Eq. S1, and the constant ratio of concentrations [P]/[m] can be removed from the sum:

$\frac{\sum_{k=1}^{N} w_{k}S_{m,k}}{\sum_{j=1}^{N} w_{j}S_{P,j}} = \frac{\sum_{k=1}^{N} w_{k}S_{m,k}}{\sum_{j=1}^{N} w_{j}{(\frac{\left[ P \right]}{\left[ m \right]}S}_{m,j})}=\frac{\left[ m \right]}{\left[ P \right]}\frac{\sum_{k=1}^{N} w_{k}S_{m,k}}{\sum_{j=1}^{N} w_{j}S_{m,j}}$ [S3]

As the weights are identical, the sums are equal and they cancel out, leaving:

$\frac{\sum_{k=1}^{N} w_{k}S_{m,k}}{\sum_{j=1}^{N} w_{j}S_{P,j}} =\frac{\left[ m \right]}{\left[ P \right]}.$ [S4]

This can be rearranged to show that [m] can be determined from the ratio of the combined signals multiplied by the concentration of the phantoms:

$\left[ m \right]=\left[ P \right]\frac{\sum_{k=1}^{N} w_{k}S_{m,k}}{\sum_{j=1}^{N} w_{j}S_{P,j}}$. [S5]

Note that this relies on the normalization factors being identical, so that Eq. S2 holds, and the weights being identical so that the sums cancel.

## Derivation of a combined sensitivity correction *η* from the Roemer formulae

Roemer et al. describe two types of coil combination: uniform noise and uniform sensitivity (1). The uniform noise spectrum with maximal SNR is given by:

$P\left( \boldsymbol{r,}\delta\right)=C\frac{{\hat{\boldsymbol{p}}\left( \boldsymbol{r,}\delta\right)}^{T}\left( \boldsymbol{\Psi}^{-1} \right)^{*}\hat{\boldsymbol{b}}\left( \boldsymbol{r} \right)^{\boldsymbol{*}}}{\sqrt{{\hat{\boldsymbol{b}}\left( \boldsymbol{r} \right)}^{\dagger}\left( \boldsymbol{\Psi}^{-1} \right)^{*}\hat{\boldsymbol{b}}(\boldsymbol{r})}}$ [S6]

where *P* is the combined complex-valued point in voxel ***r*** at chemical shift δ, C is an arbitrary constant, $\hat{\boldsymbol{p}}$ is the column vector of points from each element, $\boldsymbol{\Psi}$ is the noise covariance matrix, and $\hat{\boldsymbol{b}}$ is the column vector of the receive field ***B***_1_^-^ for each element. ^T^ denotes the transpose, ^*^ the conjugate, and ^†^ the conjugate transpose.

The noise covariance matrix can be measured robustly for a 16 element array from > 10^5^ noise samples$\hat{\mathbf{n}}$ according to

$\boldsymbol{\Psi}\boldsymbol{=}\left\langle\left( \hat{\boldsymbol{n}}\boldsymbol{-}\left\langle\hat{\boldsymbol{n}} \right\rangle_{\mathbf{time}} \right)\left( \hat{\boldsymbol{n}}\boldsymbol{-}\left\langle\hat{\boldsymbol{n}} \right\rangle_{\mathbf{time}} \right)^{\dagger} \right\rangle_{\mathrm{time}}\boldsymbol{.}$ [S7]

To quantify metabolite concentrations, i.e. for absolute quantification, uniform *sensitivity* scaling must be used instead of uniform noise. The uniform sensitivity formula is given by:

$P\left( \boldsymbol{r,}\delta\right)=C\frac{{\hat{\boldsymbol{p}}\left( \boldsymbol{r,}\delta\right)}^{T}\left( \boldsymbol{\Psi}^{-1} \right)^{*}\hat{\boldsymbol{b}}\left( \boldsymbol{r} \right)^{\boldsymbol{*}}}{{\hat{\boldsymbol{b}}\left( \boldsymbol{r} \right)}^{\dagger}\left( \boldsymbol{\Psi}^{-1} \right)^{*}\hat{\boldsymbol{b}}(\boldsymbol{r})}$ [S8]

In order to split up this problem, Eq. S8 can be rewritten as:

$P\left( \boldsymbol{r,}\delta\right)={\hat{\boldsymbol{p}}\left( \boldsymbol{r,}\delta\right)}^{T}\boldsymbol{W(r)\eta(r)}$ [S9]

where

$\boldsymbol{W}\left( \boldsymbol{r} \right)=C\frac{\left( \boldsymbol{\Psi}^{-1} \right)^{*}\hat{\boldsymbol{b}}\left( \boldsymbol{r} \right)^{\boldsymbol{*}}}{\sqrt{{\hat{\boldsymbol{b}}\left( \boldsymbol{r} \right)}^{\dagger}\left( \boldsymbol{\Psi}^{-1} \right)^{*}\hat{\boldsymbol{b}}(\boldsymbol{r})}}$ [S9]

is the vector of the weightings *w*(***r,****k)* of each element *k* and

$\boldsymbol{\eta}\left( \boldsymbol{r} \right)=\frac{1}{\sqrt{{\hat{\boldsymbol{b}}\left( \boldsymbol{r} \right)}^{\dagger}\left( \boldsymbol{\Psi}^{-1} \right)^{*}\hat{\boldsymbol{b}}(\boldsymbol{r})}}$ [S10]

is a vector of per-voxel sensitivity correction factors.

## Phantom size simulation

A set of cylindrical phantoms of 0.45 S.m^-1^ conductivity were simulated, independently varying the depth (between 100 and 336 mm, see Fig. S1) and radius (between 125 and 350 mm, see Fig S2).


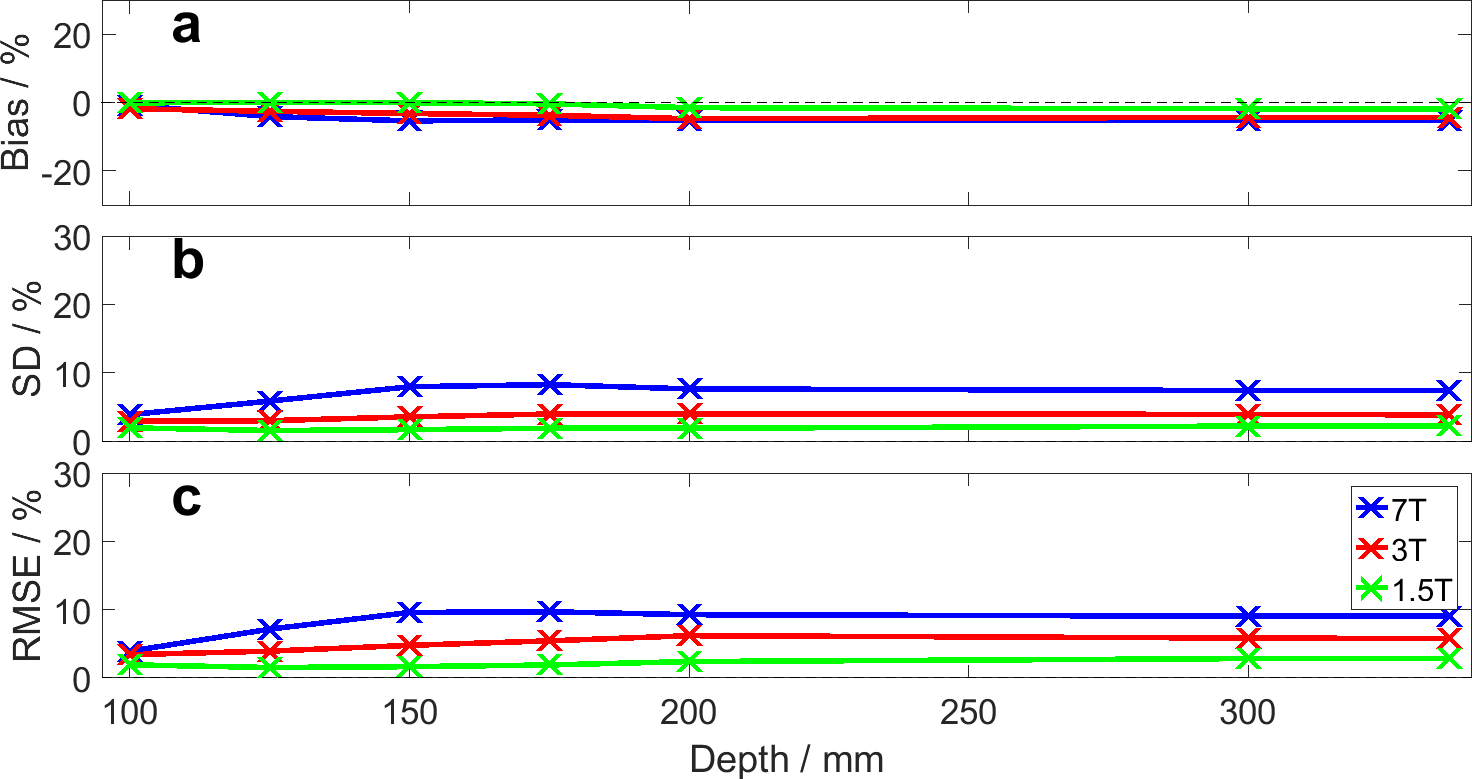


**Fig S1. B_1_ error for different depth cylindrical phantoms.**


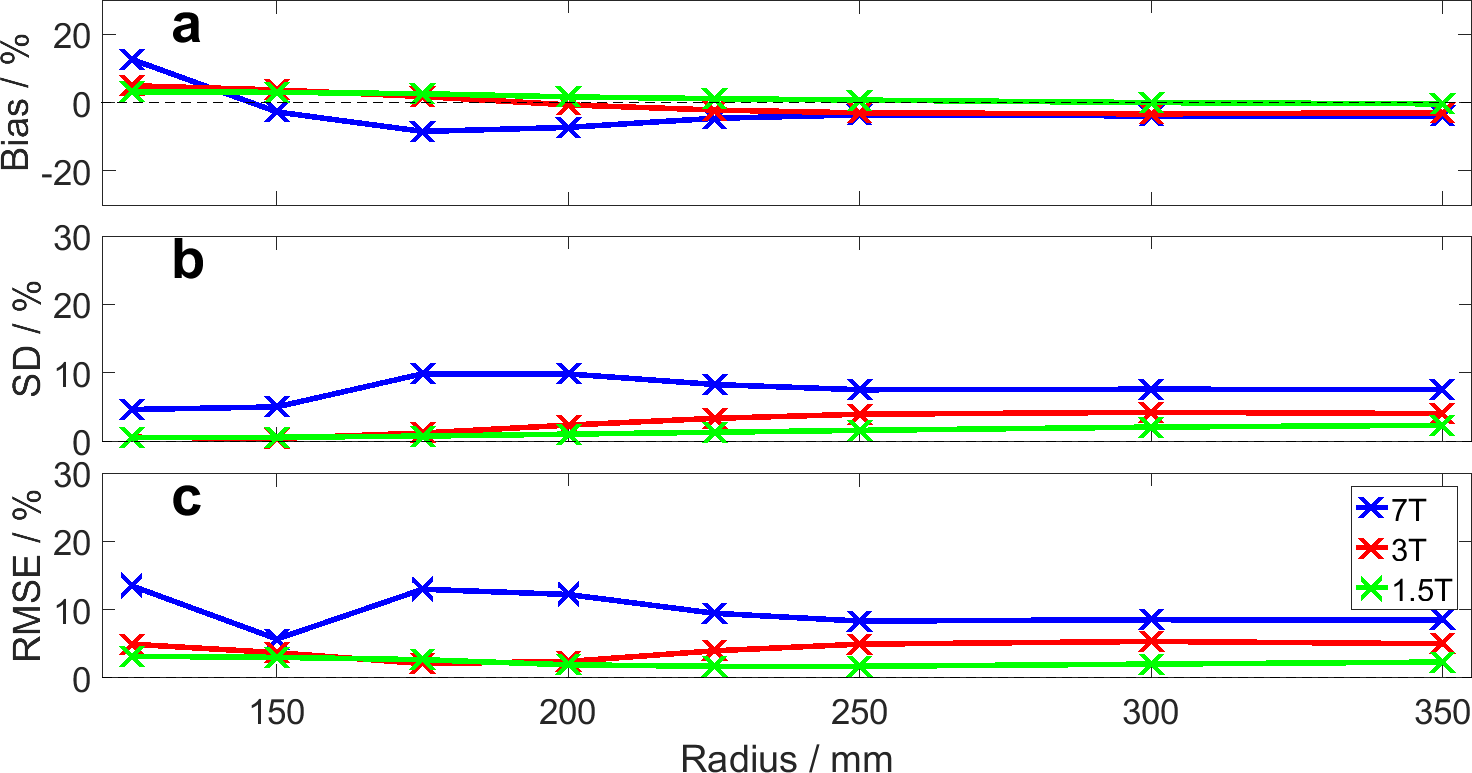


**Fig S2. B_1_ error for different width cylindrical phantoms.**

The minimum depth with consistent error (i.e. less 1% change) between points is 200 mm. The RMSE at 200 mm depth is 9.3% for 7 T, 6.2% for 3 T, and 2.4% for 1.5 T. The minimum width with consistent error is 250 mm. The RMSE at 250 mm depth is 1.7% for 1.5 T, 5.0% for 3 T, and 8.3% for 7 T.

The radius of the cylindrical phantom must be larger to have consistent errors than the depth. This is because the edges of the coil are 50 mm closer to the edge than the bottom. However, the error is lower for radius than for depth because the sides are curved, compared to the flat bottom of the phantom.

The size of the jerry can phantom was chosen to give consistent errors with simulation, rather than minimize errors. Ideally, there would be an iterative process across size and conductivity to find the perfect phantom. In practise, this is unreasonably time consuming, and relies on the simulations perfectly matching the “real world” results. It is easier to match conductivity than find a perfectly sized phantom. In addition, the perfect size varies from field strength to field strength. It is therefore better to choose a phantom size that gives consistent errors than to pick one that matches perfectly the in vivo values.

## Phosphate conductivity determination

5.5 L cylindrical aqueous potassium phosphate (K_2_HPO_4(aq)_) phantoms were made up for six concentrations: 15, 18, 20, 30, 40 and 50 mM. For each phantom, a 10 cm loop was tuned and matched to 120.3 MHz (i.e. 7 T for ^31^P). The concentration of a NaCl phantom of equal size solution was increased until the S_11_ for the 10 cm loop matched that of the K_2_HPO_4_ solutions. The conductivity was then interpolated using data from Schwan (2). Static conductivities were calculated by interpolating data from Pethybridge et al. (3) and the CRC Handbook (4).

**Table S1:** Conductivities of various phosphate solutions

| K_2_HPO_4_ concentration / mM | Static conductivity / S.m^-1^ | Conductivity at 120.3MHz / S.m^-1^ |
| --- | --- | --- |
| 15 | 0.30 | 0.32 ± 0.02 |
| 18 | 0.35 | 0.36 ± 0.02 |
| 20 | 0.38 | 0.45 ± 0.02 |
| 30 | 0.54 | 0.52 ± 0.02 |
| 40 | 0.70 | 0.89 ± 0.02 |
| 50 | 0.86 | 1.09 ± 0.02 |

The conductivity would be expected to increase to 120.3 MHz based on the Debye-Falkenhagen effect.

## Sample in-vivo spectra


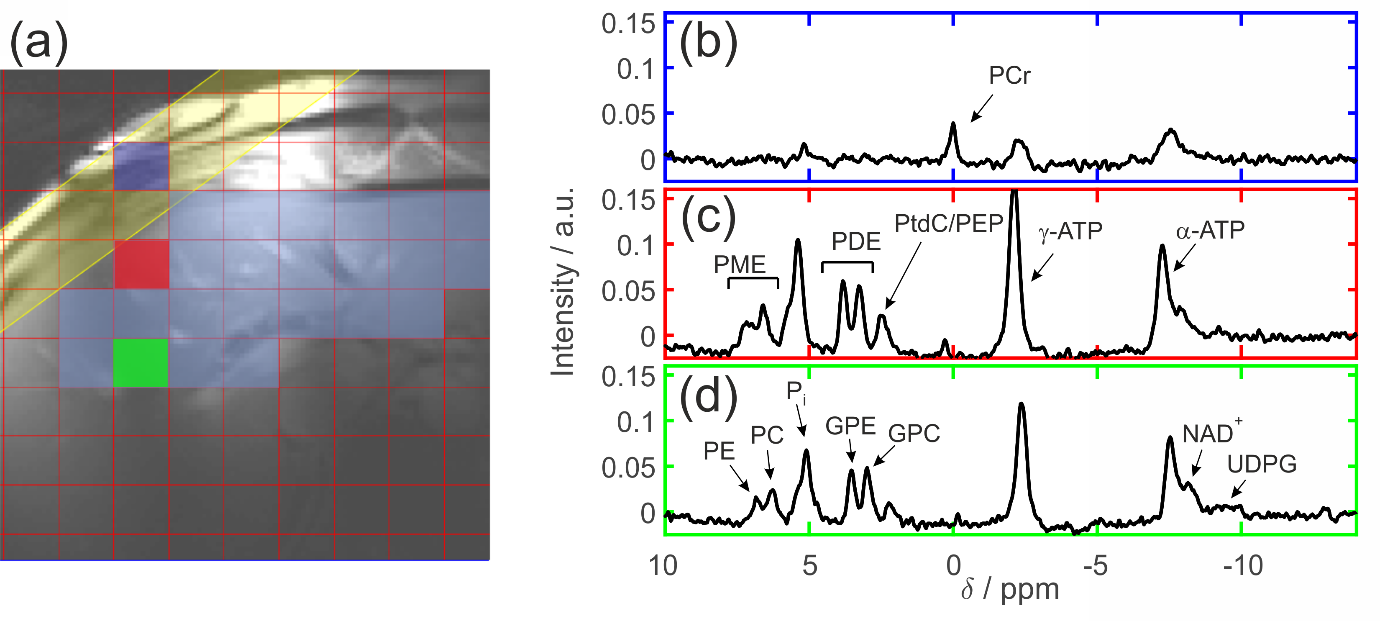


**Supporting Information Figure S3:** Sample spectra acquired from the human liver in a healthy volunteer using the 16-element array coil. (a) Transverse localiser image overlaid with the saturation band (yellow) and CSI matrix (red). Voxels from this slice that met the quality criteria and were used for further analysis are highlighted. (Note that all high quality liver voxels from all slices were used in the analysis.) (b-d) Representative spectra from the corresponding voxels highlighted in (a). (b) is in skeletal muscle and shows PCr signal, (c-d) are from liver and show negligible PCr.

## References

1. Roemer PB, Edelstein WA, Hayes CE, Souza SP, Mueller OM. The NMR Phased Array. Magn Reson Med. 1990; 16(2):192-225.

2. Schwan HP. Electrical Properties of Tissue and Cell Suspensions. In: John H L, Cornelius A T, editors. Adv Biol Med Phys. Volume 5: Elsevier; 1957. p. 147-209.

3. Pethybridge AD, Talbot JDR, House WA. Precise Conductance Measurements on Dilute Aqueous Solutions of Sodium and Potassium Hydrogenphosphate and Dihydrogenphosphate. J Solution Chem. 2006; 35(3):381-93.

4. CRC Handbook of Chemistry and Physics. 70th ed. Boca Raton, FL: CRC Press; 1989.
